# Physical Therapists’ Use of Behavior Change Strategies to Promote Physical Activity for Individuals with Neurological Conditions

**DOI:** 10.3390/healthcare13192485

**Published:** 2025-09-30

**Authors:** Amber LaMarca, Gwendolyn Larsen, Kathleen D. Lyons, Julie Keysor

**Affiliations:** 1 Rehabilitation Sciences, MGH Institute of Health Professions, 36 1st Av., Boston, MA 02129, USA; klyons2@mghihp.edu (K.D.L.); julie_keysor@uml.edu (J.K.); 2 Department of Physical Therapy Program, MGH Institute of Health Professions, 36 1st Av., Boston, MA 02129, USA; 3 School of Health Care Leadership, MGH Institute of Health Professions, 36 1st Av., Boston, MA 02129, USA; 4 Department of Occupational Therapy, MGH Institute of Health Professions, 36 1st Av., Boston, MA 02129, USA; 5 Department of Physical Therapy, Spaulding Rehabilitation Hospital, 300 1st Av., Boston, MA 02129, USA; 6 Department of Physical Therapy and Kinesiology, University of Massachusetts Lowell, 220 Pawtucket St., Lowell, MA 01854, USA

**Keywords:** behavior change, physical activity, health promotion, neurorehabilitation, physical therapy

## Abstract

**Background/Objectives**: People living with neurological conditions are inactive despite widespread literature showing physical activity (PA) is beneficial for this population. To impact long term changes in PA behavior, physical therapists treating individuals with neurological conditions need to provide effective PA promotion in combination with behavior change techniques (BCTs). The purpose of this study is to (1) characterize the use of BCTs during neurorehabilitation, and (2) to gain an understanding of considerations related to PA promotion. **Methods**: Observations of outpatient physical therapy encounters with subsequent semi-structured interviews were conducted. Observations were transcribed with detailed field notes and analyzed with descriptive analysis and deductive coding. Interviews were analyzed with thematic analysis. **Results:** Observations indicated that PA promotion in neurorehabilitation practice emphasizes home exercise programs, with less focus on aerobic activity. The most common BCTs used were instruction on how to perform the behavior, behavioral practice and rehearsal, and social support. Primary themes that impacted physical therapist use of BCTS for PA promotion included knowledge, decision processes, perceived role, beliefs, environmental context, and social influences. **Conclusions:** PA promotion from physical therapists in neurorehabilitation is not targeting activity guidelines and there is uncertainty about using behavior change strategies and PA guidelines for PA promotion.

## 1. Introduction

Chronic neurological conditions, including stroke, Parkinson’s disease, traumatic brain injury, multiple sclerosis, and spinal cord injury are leading causes of disability globally and result in many lifelong multisystem impairments including mobility, cognition, and more [1]. Physical activity (PA) is recommended for individuals with neurological impairments for long-term management of health outcomes with an emphasis on moderate-intensity aerobic activity combined with strengthening, balance, and stretching [2,3,4,5]. PA can improve overall health factors (e.g., reducing blood pressure and arterial stiffness), improve neuroplasticity and functional outcomes (e.g., walking and balance), and reduce secondary complications and comorbidities [6,7,8,9]. However despite the well-known benefits of PA, people with neurological conditions are largely inactive [10,11,12,13]. To increase PA, it is recommended that healthcare professionals utilize behaviorally informed PA promotion, which is the use of behavior science to help guide the adoption of PA behaviors [14,15,16,17]. A critical component of behaviorally informed PA promotion is the use of individualized behavior change techniques (BCTs) such as goal setting, problem solving, and feedback [18]. Use of BCTs is linked to improvements in PA promotion in adults with musculoskeletal and chronic disease conditions [19,20,21,22,23,24,25].

However, despite recommendations and the widespread understanding of the importance of use of behavioral strategies to promote PA, it is rarely performed in practice by physicians and general practitioners [26,27]. Physical therapists with neurological clinical expertise may be well-poised to fill this gap as they are skilled in implementing effective treatments to address the functional needs of this population [28,29,30], and may be well-suited to promote PA when patients are being discharged from outpatient neurological physical therapy [27,31,32]. Behaviorally informed PA promotion approaches coupled with treatment of the complex physical elements of neurological conditions such as impaired motor control, muscle weakness, instability, gait impairments, and low endurance could be beneficial for patients as they transition to the home and community to promote long-term PA behavior [33,34,35,36]. Yet, little is known regarding how BCTs can be used to promote PA in adults with chronic neurological conditions. To our knowledge, there are no studies that explore how physical therapists in outpatient neurological clinics use behaviorally informed approaches to promote PA to help transition patients from outpatient clinical care to home-based PA programs.

The purpose of this study was to (1) characterize PA promotion and the use of behavior change strategies during physical therapists’ outpatient clinical encounters with people with chronic neurological conditions, and (2) gain an understanding of physical therapists’ perspectives regarding promoting home-based PA as patients are discharged from skilled outpatient therapy.

## 2. Materials and Methods

This qualitative study is reported following the Standards for Reporting Qualitative Research (SRQR) [37]. This study was approved by the local IRB (#2024P003290) and all participants provided consent.

### 2.1. Qualitative Approach and Research Paradigm

The study was a qualitative descriptive study designed with an interpretivism epistemological stance to gain an understanding of physical therapists’ actions, beliefs, motivations, and reasoning regarding promoting PA when transitioning patients from skilled care to independent home programs [38,39,40]. The overarching framework used to guide this work was the Capability, Opportunities, and Motivation System of Behavior (COM-B) [41]. The COM-B describes that there are three main components that influence behavior, capability, opportunity, and motivation, that can be further broken down into fourteen subdomains using the Theoretical Domains Framework (TDF) [42]. For a visual representation, please see Appendix A [43]. The domains of the TDF include ‘Knowledge’, ‘Skills’, ‘Social/Professional Role and Identity’, ‘Beliefs about Capabilities’, ‘Optimism’, ‘Beliefs about Consequences’, ‘Reinforcement’, ‘Intentions’, ‘Goals’, ‘Memory, Attention and Decision Processes’, ‘Environmental Context and Resources’, ‘Social Influences’, ‘Emotions’, and ‘Behavioral Regulation’. The COM-B and TDF integrated frameworks have been used to investigate healthcare professionals’ use of PA promotion in practice [44,45,46,47] and were used to guide study design, interview structure, and data analysis for this study.

### 2.2. Research Team Characteristics and Reflexivity

The research team comprised physical and occupational therapists and included individuals with backgrounds in clinical care and qualitative research. Three out of the four (AL, GL, and JK) had outpatient neurorehabilitation experience representing over 40 years of neurorehabilitation practice. AL and GL currently treat individuals with neurological conditions in the outpatient setting while engaging in academic research. Two members (JK and KDL) are experienced clinical researchers with expertise in qualitative research. Two members (AL and JK) completed the Behavior Change Taxonomy training and certification [48,49]. One team member (AL) conducted observations and semi-structured interviews. Two members analyzed the observational data (AL and JK); two members analyzed the semi-structured interview data (AL and GL). The team member that performed the observations and interviews (AL) had previous experience working in the hospital network and knew the physical therapist participants in the study. As a result, interview responses may be impacted by the familiarity from previous working relationships and a tendency to speak towards shared experiences. Her knowledge and past experiences assisted with her understanding of the work environment, patient population, and discharge process. To complement extensive clinical experience while minimizing potential bias related to known relationships with the study participants, a pre-determined interview guide and process informed by the COM-B [50], TDF [42], and scientific evidence was employed. Additionally, reflexivity was applied by the research team by discussing and challenging assumptions throughout the data analysis process.

### 2.3. Context

As little is known about this topic, we selected a setting known for its high standard of care in neurorehabilitation as this was most likely to reflect best practice. We targeted licensed physical therapists working in a neurological outpatient practice in an internationally recognized academic rehabilitation hospital, Spaulding Rehabilitation Hospital in Charlestown, MA, who were preparing patients for discharge from skilled physical therapy. Spaulding Hospital is recognized as a leader in neurological rehabilitation in the United States and is actively involved in academic research and teaching.

Prior to the observations and semi-structured interviews, therapists were informed that the purpose of the study was to understand the strategies physical therapists utilize to transition patients from clinic to home- and community-level PA during the discharge process. Observations were completed in the natural treatment environment of the outpatient physical therapy clinic to allow for the observation of the environmental context including resources and social interactions.

### 2.4. Sampling Strategy

Convenience sampling was utilized to recruit participants (i.e., physical therapists) who worked in the neurological outpatient department. Novice and experienced clinicians were sought for inclusion. Five physical therapists were enrolled, each with two patient encounters. Inclusion criteria included adults (18 years or older), individuals who were English-speaking, licensed physical therapists that performed at least 20 h of patient care per week, maintained a caseload comprising at least 50% patients with neurological conditions, and had at least two patients on caseload with anticipated discharge in the next month. Patients that were included in observations had to have adequate cognitive function and speak English to provide verbal consent for the observation of their clinical encounter.

### 2.5. Data Collection Methods

Data collection was performed from December 2024 through February 2025. Ten observations were completed, two observations for each participant (therapist). Each therapist completed a semi-structured interview at the completion of the second observation. Therapists’ demographics (age, gender, specialty certification, years of practice) were obtained by self-report and patient demographics were obtained (age, gender, primary diagnosis, ambulation status, caregiver presence during clinical encounter) from the medical chart.

#### 2.5.1. Observations of Physical Therapist–Patient Clinical Encounters

Data were collected pertaining to physical therapist–patient communications and behaviors (AL). Discharge or discharge-planning encounters were chosen to capture conversations regarding therapist recommendations for home and community PA after discharge. Each clinical encounter was scheduled for 45 min. Efforts were made to avoid interruption of the traditional flow of clinical encounters or planned activities during observations.

Detailed field notes were taken to document the conversations, facial expressions, body language, actions, environmental characteristics, targeted PA behaviors, assessments of PA, and the context surrounding PA promotion. In addition to in-the-moment field notes, the researcher completed expanded detailed notes immediately after the observations. The researcher also completed a same-day debriefing session with physical therapists to allow the therapist to briefly share additional context of the patient’s situation, the clinical encounter that was observed, and their thoughts on the clinical encounter. The debrief was typically about 5 min in length or as time allowed based on the therapist’s schedule. Audio/video recordings were not plausible due to the open settings of the gym. Following observations, a reflective memo was completed that summarized the researcher’s evolving reflections and interpretations of the data.

#### 2.5.2. Semi-Structured Interviews

Semi-structured interviews were conducted, recorded, and transcribed via a secure Zoom platform. All interviews were conducted using a pre-defined interview guide by one investigator (AL). The order of questions asked was variable based on conversation flow. Additional prodding questions were employed to expand on each participant’s experience. Whenever possible, open-ended questions were asked to study participants to elicit data in a non-biased manner.

The priorities of the interview were to (1) triangulate data from observations by asking therapists about what PA promotion strategies they use in practice and (2) understand capabilities, opportunities, and motivations that impact BCT use for PA promotion. First, therapists were asked about strategies they used for PA promotion. Second, they were asked questions based on the TDF model to address the subdomains of capabilities, opportunities, and motivations behind BCT use for PA promotion. See Appendix B for interview guide.

### 2.6. Data Processing and Analysis

#### 2.6.1. Observations of Physical Therapist–Patient Clinical Encounters

After observations were completed, field notes were further revised, expanded, and cleaned for interpretation and descriptive statistics were computed. BCTs were coded according to the Behavior Change Technique Taxonomy [48,49].

First, the target PA behaviors were identified for each field note (AL). Target PA behaviors had to meet two criteria to be included: (1) it was classified as PA, defined using the older adult and adult wheelchair user physical activity compendiums [51,52], and (2) it was intended for carryover to the home or community setting. PA behaviors were then classified by the type of PA (e.g., walking activity, resistance exercise, recreational activity, household activity, aerobic exercise). While many physical activities could be considered aerobic, we used the code “aerobic exercise” only when the PA prescription was given with explicit instructions to focus on elevating one’s heart rate or reaching a higher level of perceived exertion.

Second, deductive coding of field notes [53] was performed using the predetermined Michie’s Behavior Change Technique Taxonomy [48] to code specific BCTs during the observed clinical encounters. Two independent researchers (A.L. and J.K.) extracted field note text that included promotion of target PA behaviors and then applied a corresponding taxonomy code.

Third, the coders extracted how BCTs were carried out (verbal instructions, use of materials and handouts, etc.), if PA parameters were prescribed (i.e., frequency, duration), and whether the strategy was initiated by the therapist, patient, or caregiver. To evaluate agreement, the average interrater agreement was calculated for BCT coding between observations and across item level for each BCT code.

#### 2.6.2. Semi-Structured Interviews with the Physical Therapists

Interview recordings were transcribed via Zoom then cleaned, de-identified, and verified for accuracy alongside audio recordings. Filler words were also removed from transcripts (“like”, “um”, “kind of”, etc.) to enhance clarity for analysis. Two coders (AL and GL) reviewed the transcripts to identify relevant content for further analysis.

The portion of the interview used for triangulation of observations was deductively coded by two coders (AL and GL) for strategies that therapists reported they used for PA promotion. This was compared with BCTs that were observed during clinical encounters.

Additionally, the coders (AL and GL) explored therapist perspectives on BCT use for PA promotion. The coders inductively coded interviews and created operational definitions that were kept in a code book. After every transcript was analyzed, the coders met to discuss and iteratively create and modify the codes. If coders disagreed on a code, it was discussed until a consensus was formed. The coders then looked for patterns and created themes [54,55]. To guide interpretation and to ground the results in theory, the themes were located within TDF domains using TDF domain definitions [42,50]. Throughout, the researchers made conscious efforts to reflexively question their own preconceptions and assumptions.

### 2.7. Techniques to Enhance Trustworthiness

For observations, a debriefing session was performed for member checking after each clinical encounter to fill in gaps and clarify what was observed during the clinical encounter [56]. Triangulation of the data was performed by asking therapists about PA promotional strategies during the interviews as well. A clear audit trail was performed throughout the research study, including research memos after each observation to document reflections and interpretations of the data as it was collected. Coding decisions and rationale were also documented throughout the coding process. Two coders were used to discuss and challenge assumptions and interpretations of codes.

## 3. Results

### 3.1. Study Participant Characteristics

Five physical therapists were each observed in two separate clinical encounters with different patients (*n* = 10 observations). Interviews were conducted after completion of both observations (*n* = 5). The participants were primarily female (80%), 25–34 years of age, and had over 5 years of experience (See Table 1). The patients treated by the therapists were primarily male (60%), recovering from a stroke (50%), and used an assistive device (40%). The mean age was 60.5 years of age and half had a caregiver present during the session (See Table 2).

### 3.2. Agreement Data

Interrater agreement after coding the first four observations was moderate (60%). To improve agreement, the two coders met and modified guidelines for coding to predefine the target activity behaviors for each observation and limit codes to apply towards promotion of home activity only. Coding was redone after adjustments and the resulting average interrater agreement of BCT coding between observation was 97.8% (range 92.4–100). Percent agreement was also calculated for each behavior change code used with average agreement of 91% and range of (70–100).

### 3.3. Aim 1: Characterization of BCT Use During Observations of Clinical Encounters

#### 3.3.1. Observations

In total, a target activity behavior was promoted 35 times. The most frequently targeted home PA behaviors were resistance training with a home exercise program (14/35 40%) and walking activity (7/35 20%). Only (4/35 11%) of targeted behaviors were promoting aerobic activity. Specific activity prescription (including reps, frequency, duration, etc.) was performed more frequently when providing home exercise program printouts that were targeting resistance exercises (3/35), but infrequently for walking activity and aerobic activities (1/35). BCTs for PA promotion were largely carried out via conversations between the therapist and patient/caregiver.

**Figure 1:**
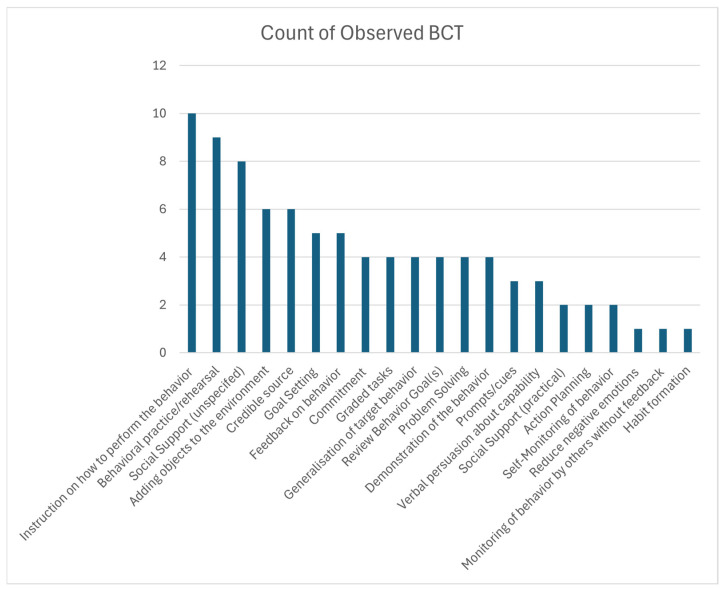
Behavior change technique use targeting home and community PA promotion.

Out of the BCT taxonomy, 27/93 types of BCTs were observed across clinical encounters. There were 80 instances of BCTs observed. The average BCTs used per clinical encounter was 8.0 techniques (range 4–15). The most common BCTs used across all clinical encounters were “instruction on how to perform the behavior” (10/10 encounters, 100%), “behavioral practice and rehearsal” (9/10 encounters, 90%), “social support” (8/10 encounters, 80%), “credible source” (6/10 encounters, 60%), “adding objects to the environment” (6/10 encounters, 60%), and “goal setting” (5/10 encounters, 50%) (Figure 1, Table 3). There was another technique noted that did not fit the BCT Taxonomy; however, the coders felt it was applied as strategy to promote the behavior. This prompted an additional code “behavior check-in for accountability”. “Behavior check-in for accountability” (6/10 encounters, 60%) was a strategy employed by therapists when they asked a patient if they had performed an activity at home. This was a verbal check-in and did not include any feedback, support, instruction, or monitoring and thus did not fit the taxonomy.

#### 3.3.2. Behavior Change Strategies Reported During Interviews

Therapists were asked to describe strategies they used to promote PA. The strategies reported by the therapists largely coincided with observations. A total of 21 strategies were noted by therapists. Education was the most prominent strategy noted in every interview (5/5) by physical therapists which was coded in observations as “instruction on how to perform behavior” and “credible source”. Other prominent strategies that therapists reported using were building social support/relationships (4/5) and goal setting (4/5). These were observed in clinical encounters and coded as “social support” and “goal setting”. Therapists discussed using activity monitoring informally in interviews. However, this type of monitoring did not meet the qualifications of a true behavior change strategy as it was not specific or logged by the patient and, therefore, did not occur frequently in coding. Also, all therapists brought up using an individualistic approach by promoting activities that are salient and enjoyable to the patient. This approach did not have a direct BCT code to link to. A less prominent but identified strategy was incorporating exercise into daily routines (2/5) which included behavior substitutions and using visual cues to exercise. These approaches were present but not consistently used in observations. Lastly, “behavioral practice and rehearsal” and “adding objects to the environment” were prominent in clinical encounters but were not brought up as explicit strategies in interviews. A table indicating how therapist reported strategies mapped onto observed strategies can be seen in Table 4.

### 3.4. Aim 2: Physical Therapists’ Perspectives Regarding Promoting Home-Based Physical Activity After Discharge from Outpatient Services

**Figure 2:**
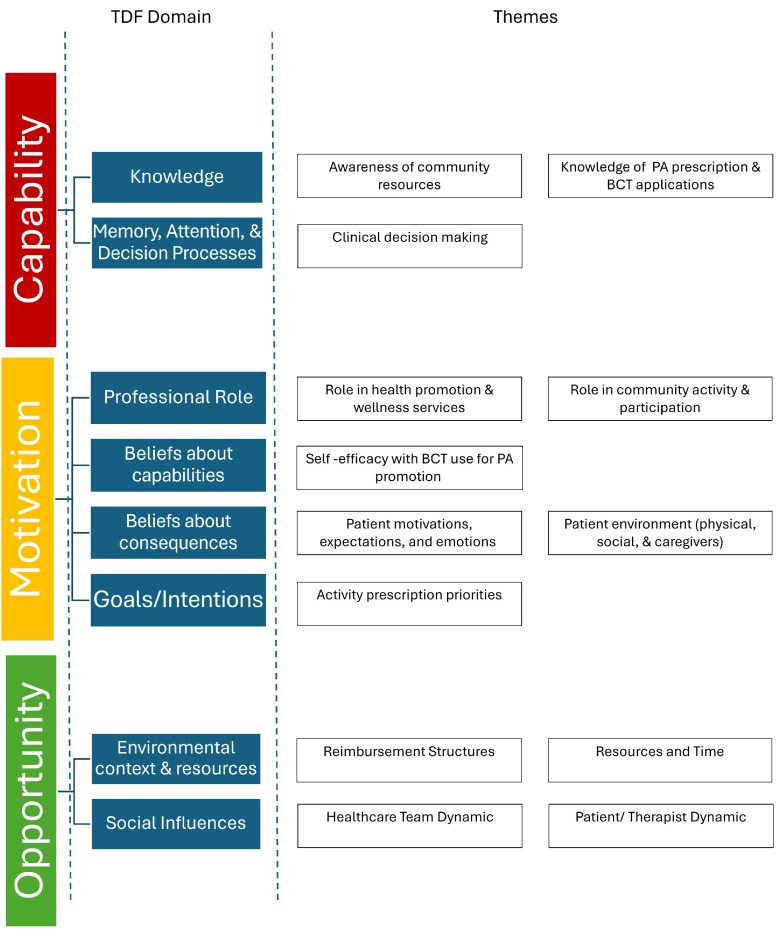
Themes mapped onto the Theoretical Domains Framework and COM-B system.

Thirteen themes were generated for therapists’ perspectives regarding promotion of PA in the home/community setting that mapped onto eight TDF domains: (i) knowledge, (ii) memory, attention, and decision processes, (iii) professional role, (iv) beliefs about capabilities, (v) beliefs about consequences, (vi) goals/intentions, (vii) environmental context and resources, and (viii) social influences. Table 5 represents a summary of themes and relevant quotations. The TDF domains mapped onto all three components of the COM-B model. For a visual representation of themes and the mapping onto the COM-B and TDF, refer to Figure 2. For further information and a visual of coding trees, please refer to Appendix C.

**(i)**  **Knowledge:** Two themes aligned with knowledge: (i) awareness of community resources and (ii) knowledge of PA prescription and BCT applications.
  a. **Lack of awareness of community resources:** Participants noted a lack of awareness of up-to-date community resources and referrals, particularly for patients living in areas unfamiliar to the participants. While the hospital network did have options for referrals such as adaptive sports or community gyms, therapists expressed that options for referrals are often spread by word of mouth. Overall, therapists share the perspective that there is a lack of knowledge regarding up-to-date, accessible, and specialized services as referral sources to promote PA after discharge.
  b. **Limited knowledge of PA prescription and BCT applications:** Therapists noted their knowledge of population-specific PA prescription was limited. While therapists mentioned general guidelines (American Heart Association or CDC), these did not always seem suitable for the neurological population. Furthermore, while therapists reported using general strategies to promote PA during practice (e.g., education, social support, goal setting), their knowledge regarding behavior change and specific techniques was limited.
**(ii)**  **Memory, Attention, and Decision Processes:** One theme aligned with this domain, clinical decision-making.
  a. **Clinical decision-making impacts how PA is promoted:** Participants discussed using information regarding patients’ diagnosis, body systems (e.g., cognition, strength, balance), activity level (e.g., community ambulator), and participation (e.g., social roles) to guide recommendations of PA in the home/community setting. Impairments that impacted safety and fall risk such as balance and cognition were viewed as concerns when promoting PA that would impact the level of difficulty and type of PA promoted. Acuity of diagnosis impacted how PA promotion was approached. For example, three of the therapists mentioned they believed their patients experienced fear and anxiety regarding managing a new injury or diagnosis independently, which is heightened when discharging from outpatient physical therapy as the last structured rehabilitation after an acute injury or event. This makes it more important to promote an independent activity plan at home to help manage this anxiety. This differs from the approach to individuals with chronic conditions, where it is expected they are already managing more independently.
**(iii)**  **Professional role:** Two themes were linked to professional role, (i) views on the physical therapist role in health promotion and wellness services and (ii) views on the physical therapist role in community activity and participation.
  a. **Physical therapy has a limited role in health promotion and wellness:** Therapists all recognized the importance of activity in this population and the increased risk of inactivity and secondary consequences that individuals with neurological conditions have. However, the majority had the perspective that engaging in prolonged PA in the home and community was an unskilled service and/or fell under the role of personal trainers.
  b. **PT role is for education not motivation for community activity and participation:** All therapists felt that it was part of their role to help their patients with community integration, and it was connected to a sense of fulfillment that therapists had in their profession in two of the interviews. Community integration might include things such as returning to work, socializing, or getting involved with community groups. While this did occasionally have an overlap with increasing activity levels, it was not directly related. In terms of promoting community or home-based activity, all therapists felt it was part of their role to educate patients on activities and provide resources to be active. However, therapists put responsibility for the motivation and follow-through of these activities on the patient.
**(iv)**  **Beliefs about capabilities:** There was one theme regarding beliefs about capabilities, which was self-efficacy with BCT use for PA promotion.
  a. **Limited self-efficacy:** Physical therapists did not feel confident in their ability to successfully utilize BCTs to motivate patients to be more active. In general, they felt they were unsuccessful in their attempts to do this in practice.
**(v)**  **Beliefs about consequences**: Two themes mapped onto beliefs about consequences including (i) beliefs about how patient motivations, expectations, and emotions could impact the results of PA promotion and (ii) beliefs about how the patient environment (physical, social, and caregiver) could impact the results of PA promotion. There was a belief that barriers in these two domains would have negative consequences on efforts for PA promotion.
  a. **Belief that patient motivations, expectations, and emotions impact the results of PA promotion**: Most (*n* = 4) physical therapists felt that patients who were not motivated were “challenging”, and it was very difficult or not possible to change a patient’s exercise behaviors. Therapists also mentioned that when patients have unrealistic expectations for recovery, this can hinder the ability to promote activities that are achievable.
  b. **Belief that the patient environment (physical, social, and caregiver) could impact the results of PA promotion**: All therapists noted that logistical and environmental barriers make it challenging to encourage exercise. Cost, geographic location, transportation, and time impacts access to activity programming. Inconsistency and limited availability of PCAs is also a consideration as many community activity centers, such as gyms and adaptive programs, require a person to assist with transfers. These cumulative logistical barriers contribute to the challenge of promoting PA in this population.
**(vi)**  **Goals/Intentions:** One theme of goals and intentions was identified, which was therapist activity prescription priorities.
  a. **Activity prescription priorities impact type of PA promoted:** The goal of activity prescription was variable across participants. Some participants (*n* = 3) reported the goal for PA after discharge was to promote a home exercise program while others (*n* = 2) felt it was more important to emphasize community integration. One therapist felt it was important to provide clear activity guidelines, while the remainder encouraged patients just to be active in some capacity.
**(vii)**  **Environmental context and resources:** The themes of reimbursement structures and resources and time mapped onto this domain.
  a. **Limitations due to reimbursement structures:** Most of the therapists had the impression that using treatment time for PA and wellness-based treatments was non-billable or non-skilled (*n* = 4). Therapists felt that insurance coverage dictates why this is not performed in practice.
  b. **Limitations due to resources and time:** Most therapists (*n* = 4) reported that limited time was a barrier, both in finding time amongst many competing priorities for this complex patient population and the amount of time it takes to have difficult conversations surrounding motivation. Additionally, the availability of programs and skilled trainers to refer patients to also impacts the ability to promote PA to patients. Even in a large hospital-based clinic, the majority of therapists (*n* = 4) reported that finding resources that are accessible, affordable, and have the proper specialized equipment and care can be challenging to come by. Conversely, one therapist reported that within her hospital network, she felt lucky there were resources available to her patients.
**(viii)**  **Social influences:** Two themes mapped onto social influences, (i) healthcare team dynamics and (ii) patient/therapist dynamics.
  a. **Healthcare team dynamics can facilitate PA promotion:** Therapists felt that healthcare team dynamics including the referring physician or physiatrist support make a big difference in the effectiveness of PA promotion. Therapists report that consistent messaging across the healthcare team on the importance of PA, meeting PA guidelines, and support in promoting activity outside of PT was a facilitator to PA promotion.
  b. **The patient/therapist dynamic impacts PA promotion:** Therapists also reported that the dynamic between the patient and the therapist was important. When there was a good rapport and aligned expectations on activity, PA promotion was easier to perform, expressed by PT5. However, misaligned expectations can make it challenging.

## 4. Discussion

Our study is the first study of physical therapists that included direct observation of clinical encounters and in-depth qualitative interviews of physical therapists to characterize the use of BCTs for PA promotion during treatment encounters with people with chronic neurological conditions, and identify physical therapists’ perspectives regarding promoting home-based PA as patients are discharged from skilled outpatient therapy. Several important findings emerged. First, the majority of PA promotion behaviors during discharge-planning clinical encounters targeted home exercise (e.g., strength training) or walking programs (e.g., practice walking with a cane); promotion of aerobic activity was limited. Second, an average of 8 BCTs per session were used by physical therapists to promote PA, of which the most commonly used strategies were (i) “instruction on how to perform the behavior”, (ii) “behavioral practice and rehearsal”, (iii) “social support”, (iv) “credible source”, (v) “adding objects to the environment”, and (vi) “goal setting”. Lastly, physical therapists’ perspectives about promoting PA for patients with chronic neurological conditions were influenced by knowledge, clinical decision-making, views on professional role, beliefs about capabilities, beliefs about consequences, goals/intentions for PA, environmental context and resources, and social influences.

None of the therapeutic clinical encounters included recommendations of activity levels meeting Center for Disease Control and Prevention (CDC) guidelines or other evidence-based activity recommendations for people with neurological conditions. Rather, recommendations for PA typically included functional activities, strengthening, and home walking programs. This approach, while typical for neurological physical therapy treatment [57], does not meet recommended levels of PA for this patient population. Furthermore, it was noteworthy that therapists were generally not aware of recommended PA levels that were achievable for individuals with complex neurological conditions. Our findings concur with others who found physical therapists had limited knowledge regarding PA and exercise prescription for people with chronic pain; [58]; however, other settings such as cardiac rehabilitation utilize these guidelines more routinely in practice [59]. Previous work also suggests that there could be a relationship between therapist exercise behavior and their knowledge of and willingness to promote PA [60]. However, this was not found as a theme in our sample. While there is no one guideline for all neurological conditions due to the variability across conditions, the American Academy of Physical Therapy Neurology Chapter released a toolkit in 2020 detailing up-to-date evidence on exercise prescription recommendations for multiple sclerosis, Parkinson’s disease, traumatic brain injury, spinal cord injury, stroke, Huntington’s disease, and amyotrophic lateral sclerosis [61]. Toolkits such as these may help providers make PA recommendations to patients with complex conditions.

The most extensively used BCTs, “instruction on how to perform the behavior,” “behavioral practice and rehearsal,” and “social support”, are linked to behavior change [21,62]; however, other BCTs (e.g., “self-monitoring” “adding objects to the environment”, “information about consequences”, “goal setting”, “problem solving”, “action planning”, “feedback on behavior”, and “biofeedback”) that may have a greater impact for people with chronic conditions [23,63] were rarely used. Interestingly, while goal setting for impairment-based goals is a major aspect of physical therapy rehabilitation, goal setting for PA behaviors outside of the clinic occurred in only half of the observations. This difference was highlighted in the dissonance noted between therapist reported strategies versus observations. This suggests that while goal setting is used regularly to guide and justify clinical intervention, goal setting to promote behavior change was not prominently used when helping patients develop home-based PA programs. The inclusion of goal setting and other BCTs may help patients engage in activity in the home [23]. However, therapist knowledge regarding behavior change theory and BCT was limited, which may have impacted therapists’ use of specific techniques to promote behavior change in clinical encounters. Our findings concur with findings in orthopedic physical therapy practice that there is limited knowledge of how to apply BCTs [64].

Similarly to others [65,66], therapists viewed promotion of community engagement and activity as part of their role, but there was mixed opinions on what this entails. All therapists felt responsible for education on resources, but felt that it was hard to know and access the resources available for patients, especially those with increased needs, socioeconomic barriers, and rural geographical locations. After referring and education, therapists mostly felt that it was up to the patient to be accountable on follow through when resources were provided and that continued monitoring of PA was not a “skilled” billable service. However, education alone is insufficient to generate behavior change, and recommendations for PA promotion should incorporate behavior science to facilitate PA change [60]. Additionally, the American Physical Therapy Association has released guidelines that skilled maintenance care and remote monitoring are billable under Medicare guidelines and health promotion should be part of physical therapy practice [67]. Overall, the state of current practice patterns is not aligned with the American Physical Therapy Association [68] or the World Confederation for Physical Therapy [69] goals for increasing focus on PA screening and promotion as part of physical therapy practice. Work must be completed to shape physical therapist views on their role in health promotion and to improve knowledge on how this is performed effectively. A health-focused physical therapy model (HFPTM) has been suggested for implementing health promotion in physical therapy practice, which highlights the need for integration of behavior change interventions [18]. This suggests that to change health behaviors such as PA, physical therapists need to be able to identify target domains of behavior that can be changed, apply effective behavior change strategies to these domains, and evaluate the clinical impact of behavior change strategies. However, the findings in this study suggest physical therapists lack the knowledge, training, and tools to follow these steps. This body of work should contribute to development of physical therapy trainings, continuing education, and curriculum to emphasize PA prescription for specialized populations, use of behavior change strategies and theory in practice, and the role of physical therapists in health promotion. Future research will also need to develop valid and sensitive outcome measures to evaluate and test BCTs as well as investigate the best methods for training and implementation into practice. Additionally, new care delivery models that can be delivered in the home setting to provide long-term behavior change strategies should be considered. Embedding technologies such as remote monitoring combined with behavioral consultation into maintenance care models could be considered to bridge this care gap [70,71].

Limitations: Our study has several limitations that are worthy of consideration. First, the sample is a small, convenience sample comprising therapists from one setting. This limitation in sample size may have led to missed perspectives or incomplete saturation. However, the setting in the study was from a large, academic hospital with extensive resources and training for staff which would suggest that the targeted setting would be representative of best practice for this population. In addition, two observations of different patients were observed for each therapist (*n* = 10), which provided repeated opportunities to observe therapists across patients. Nonetheless, the small sample may not be representative of the broader neurological outpatient setting. Second, the communications of physical therapists with their patients were not audio-recorded and the field note data collection approach may have missed data. Third, only one researcher observed the clinical encounter and recorded the field notes. It is is possible that the field notes reflected the biases of the observer; however, the observer was a clinician with expertise in this patient domain, so the investigator also brought strength in understanding clinical practice patterns.

## 5. Conclusions

The findings from this study indicated that an average of 8.0 BCTs were used during each session and the application of BCTs in neurorehabilitation was mostly applied towards home exercise programs. Specific guidelines and activity prescription were rarely applied to aerobic exercise. “Instruction on how to perform the behavior”, “behavioral practice and rehearsal”, and “social support” were noted in almost all the clinical encounters. However, key behavioral approaches that are linked to successful long-term change in PA behavior such as “self-monitoring”, “goal setting”, “problem solving”, “action planning”, and “feedback” [23] were used less than 50% of the time. From the interviews, we identified factors pertaining to domains of knowledge, decision process, professional role, beliefs about capabilities, beliefs about consequences, goals/intentions, environmental context and resources, and social influences that impacted BCT use for PA promotion [64]. This study was the first to characterize BCT use by PTs working with individuals with neurological conditions and highlighted the need for increased education and implementation efforts to integrate behavior change, PA prescription, and health promotion into routine practice. Targeted education, training, resources, and implementation efforts should be made to encourage physical therapists to use effective behavior change strategies that target long-term change in activity behaviors [72].

## Figures and Tables

**Table 1:** Participant characteristics.

Therapist	Gender	Age (Years)	Specialty Certifications	Years of Experience
PT 1	F	34	NCS	5
PT 2	F	41	NCS	15
PT 3	F	29		4
PT 4	M	32	NCS	7
PT 5	F	54		34

F, female; M, male; NCS, board-certified in neurologic physical therapy.

**Table 2:** Patient characteristics.

Patient	Therapist	Gender	Age (Years)	Diagnosis	Ambulation Status	Caregiver in Session
O1	PT1	M	66	SCI	Primarily w/c user	Yes
O2	PT2	F	62	Stroke	Walks with AD	No
O3	PT4	F	72	Stroke	Walks with AD	Yes
O4	PT4	F	32	SCI	Primarily w/c user	No
O5	PT3	M	81	PD	Primarily w/c user	Yes
O6	PT3	F	49	MS	Walks with AD	No
O7	PT1	M	59	Stroke	Walks without AD	Yes
O8	PT2	M	68	PD	Walks without AD	No
O9	PT5	M	62	Stroke	Walks with AD	No
O10	PT5	M	54	Stroke	Walks without AD	Yes

MS, multiple sclerosis; PD, Parkinson’s disease; SCI, spinal cord injury; AD, assistive device; w/c, wheelchair.

**Table 3:** Behavior change technique code and examples.

BCT	Example from Field Note
Instructions on how to perform the behavior	The physical therapist brings up the documents she gave to them and repeats the instructions again for the exercise. “See how [the] walker is starting to move out, here is how you bring the walker back towards you”—(PT1, O1)
Behavioral practice and rehearsal	The therapist says, “let’s practice some [exercises] that I think would be good for you.”—(PT4, O3)
Social support	He encourages her to return to the rowing program—(PT4, O4)
Credible source	“I have a question for you though, which exercise is the one I’m the weakest at? What should I work on [at home]?” The therapist says “the balance”—(PT5, O10)
Adding objects to the environment	“You see brace clinic next week. When you get the brace, you will use it at home and it will help”—(PT3, O6)
Goal setting	The therapist asks “Do you have any new goals that we should work on?” and the patient reports he wants to play a round of golf—(PT2, O8)

BCT, behavior change technique.

**Table 4:** Therapist-reported strategies compared to observations.

Therapist Reported Strategy	BCT Used in Observation
Education (5/5)	“Instruction on how to perform the behavior” (10/10) “Credible source” (6/10)
Building Social support/relationships (4/5)	“Social support” (9/10)
Goals and planning (4/5)	“Goal setting” (5/10)
Personalization (5/5)	No associated BCT code
Behavior substitution (1/5)	“Behavioral substitution” (1/10)
Adding visual cues for exercise (1/5)	“Prompts and cues” (3/10)
Not in interview	“Behavioral practice and rehearsal” (9/10)
Not in interview	“Adding objects to the environment” (6/10)

**Table 5:** Therapist considerations for PA promotion.

TDF Domain	Theme	Example(s)
Knowledge	Lack of awareness of community resources/referrals	“I would like more geographical information or [a] flyer or handout for us to access. You know, new up-and-coming established programs. Because I think a lot of it is hearsay. There’s an email thrown out like here or there” (PT2) “I often just tell people like, ‘Go find a personal trainer’ [but] I don’t have names.” (PT4)
	Limited knowledge of PA prescription and BCT applications	“Do you know any resources as far as exercise, prescription recommendations for the neuro population that are good? The American Heart Association [has] recommendations for exercise, but those are so out of reach, for most of my people.” (PT4) “Behavior change… I remember doing a health promotion project during PT School, where I looked at a specific model, and I can’t [remember]. I’m sure it probably influences some of what I do but could I tell you what it is?” (PT1)
Memory, attention, and decision processes	Clinical decision-making impacts how PA is promoted	“I have identified a cognitive impairment, [so] I didn’t want to give him anything super hard to follow… So, the exercises I gave him [are] really basic…I just kind of thought about what would be easy enough for him to do…. or another patient, she has MS and her balance is pretty inconsistent. I want to give her something that’s not going to fatigue her too much. So that’s been kind of something I’ve kept in the back of my mind” (PT3) “I think the fear is from a patient perspective, the fear will always be that this is the last leg of rehab [ilitation] outpatient, and then I’m discharging you. ‘Oh, my goodness, am I going to be just left, hanging… like I don’t know what to do.’…. And so, releasing that hand a little slowly, would be a good idea if they are acute patients and if they’re chronic, of course they already know [what to do]. So I think that is also important to understand that in our patients [this] is the last leg of their rehab journey for the acute patients. And the discharge plan, you already think of where are they going to continue [activity]’”(PT5)
Professional Role	Physical therapy has a limited role in health promotion and wellness	“Because then I’ve discharged them. It’s not something that they are on my program for me to follow up in the gym. Then that’s left to the gym instructors. That’s a different role” (PT5)
	PT role is for education, not motivation for community activity and participation	“I think we should view the continuity of care not ending an outpatient. I think it needs to be about what’s then the plan for people being integrated in communities. And it’s not just ‘are they meeting the physical activity guidelines?’ Yes, they should. But are they able to participate in society? Are they able to work? Are they able to go to a restaurant? Are they able to go to a gym?” (PT1) “[I am] providing and educating on examples of opportunities that they can take part of, but we talk about ultimately there’s only so much I can do, It’s kind of on them. And I try to make that expectation of them doing their part.” (PT2)
Belief about capabilities	Limited self-efficacy	“I don’t know if I’m good at it, to be honest. I feel like I should take some community courses on it. It’s actually on my list of things to improve on cause yeah, I work hard at doing a day of motivational interviewing with people and trying to have them figure out how to make themselves better. …But I don’t know if I’ve been successful at it.” (PT4)
Belief about consequences	Belief that patient motivations, expectations, and emotions impact the results of PA promotion	“People are the same people they were before the injury or after the injury, like if they weren’t motivated to exercise before they’re not motivated to exercise afterward” (PT3) “You know, those are the more challenging patients to get into a routine…. The expectations that some patients have who have neuro diagnoses are sometimes a little bit far-fetched.” (PT2)
	Belief that the patient environment (physical, social, and caregiver) could impact the results of PA promotion	“It’s a production… [it] takes all day for them to do any activity. So for them to go to the gym [or] to meet a personal trainer for half an hour… Yes, they were doing that for me but that is a healthcare thing. But when it’s not, and now they’re paying for the ride, they’re paying for the personal trainer, they’re spending half the day doing it, and [they’re paying for] their PCA… so it becomes a whole ordeal where it takes them a whole day to go to the gym. So then they don’t…. so this is probably my biggest challenge, my biggest barrier” (PT4)
Goals/intentions	Activity prescription priorities impact type of PA promoted	“My goal would be to have them have like a routine and exercise program for them to continue on outside of this space.” (PT2) ‘I definitely am less focused on the exercise as a prescription and more focused on the exercise as a tool for activity and like to do life.” (PT4)
Environmental context and resources	Limitations due to reimbursement Structures	“I always have always been a huge advocate for creating [activity] programs. Unfortunately, now, it’s so insurance [and] productivity driven that we cannot bill these patients.” (PT5)
	Limitations due to resources and time	“But having those conversations was quite challenging. And so I spent a lot of effort doing it…it’s a time thing. it is the setting aside the time to have the difficult conversation” (PT4) “For people who need specialized equipment, I would love to be able to send everyone to an affordable, geographically accessible, adaptive gym, where there’s a standing frame that you can use…. I think, just like in the able bodied, or non-disabled population, it’s a question of access, ease, geographic access, money. It’s going to be the same in this population. But there’s less options [to refer to].” (PT1) “I don’t know about everyone else, I can only say for myself, my patients have the resources. I mean, I hope everyone does. We’re lucky we’re here…. At [our hospital], we have the adaptive sports program…we have a lot of groups, there’s support groups here.” (PT5)
Social influences	Healthcare team dynamics	“I think I’m also lucky, like one of the physiatrists… He also often makes that [physical activity] a recommendation too. So, I think we both see each other’s notes, and we’re like, “Oh, Hey, cool!” And it’s nice having people on the same page.” (PT1)
	Patient/therapist dynamic	“So you’re interacting with them a lot. So you build up that rapport where you know this motivates them [or] that doesn’t motivate them. …. So you know, coming to therapy motivates them [to be active].” (PT5) “She had a massive aneurysm, and she always wants to walk every single time she’s here, but couldn’t really walk… the whole PT Episode, she perseverates on wanting to walk but she can’t. So we never actually get anything done because walking, was never really a reasonable goal for her…. and the discharge for her has always been really emotional for her and her mom, and everybody’s really upset” (PT4)

TDF, Theoretical Domains Framework; MS, multiple sclerosis.

**Figure A1:**
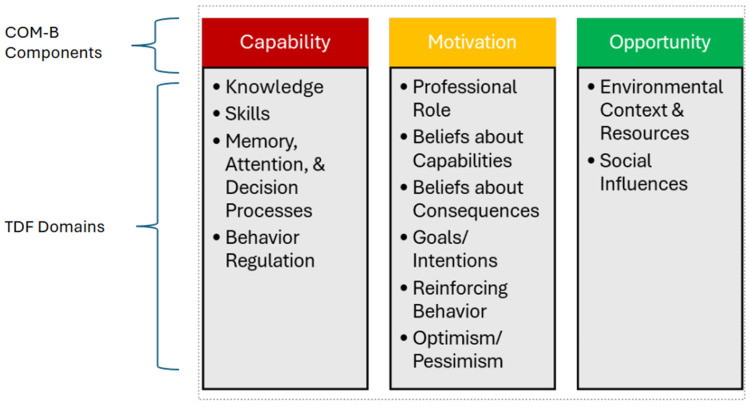
Adapted from the Capability, Motivation, Opportunity System and Theoretical Domains Framework Model by the Center for Implementation 2023.

**Figure A2:**
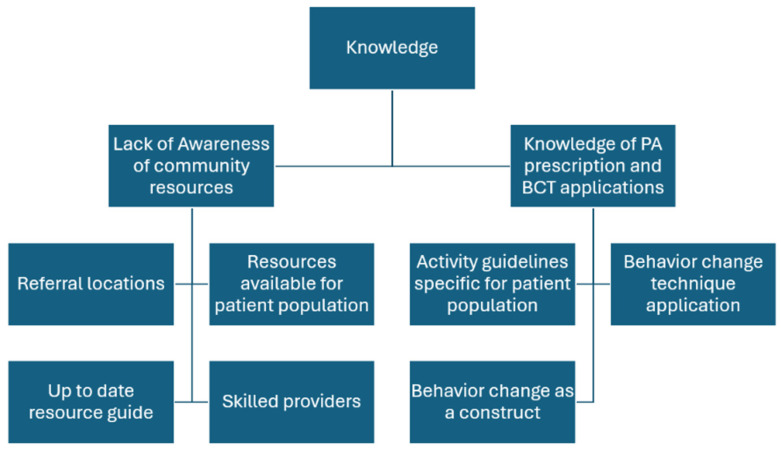

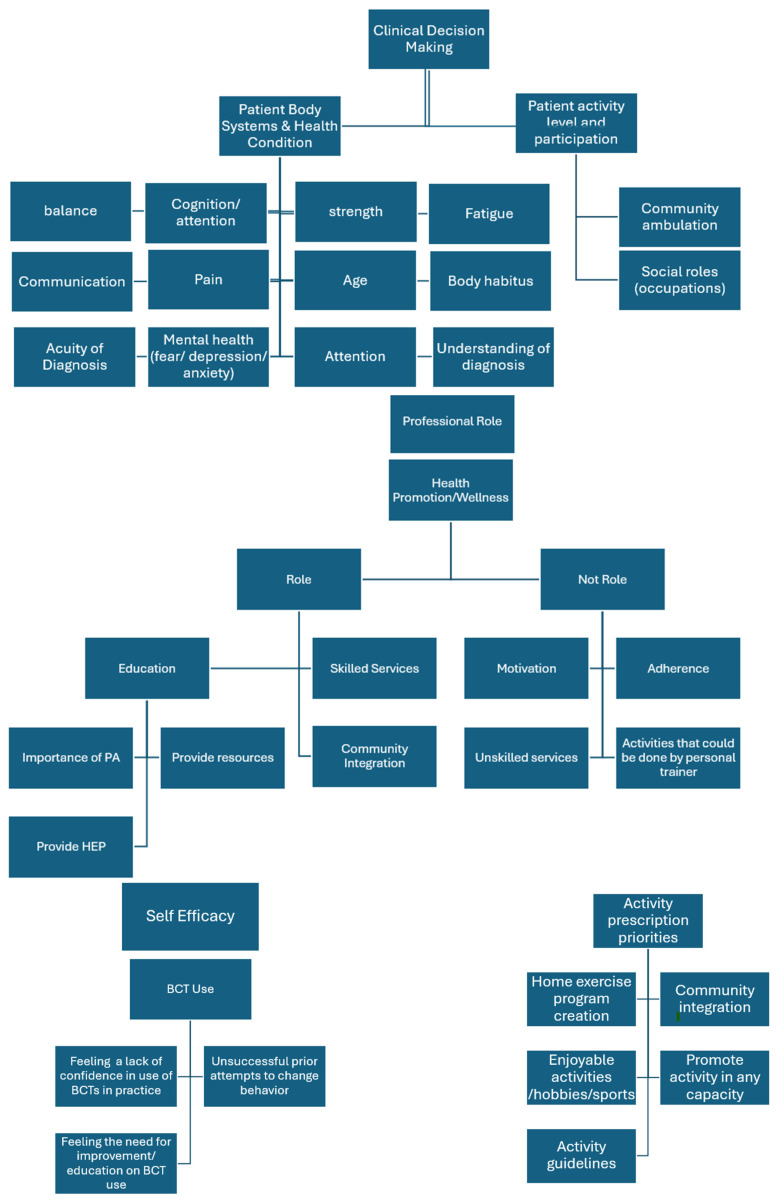

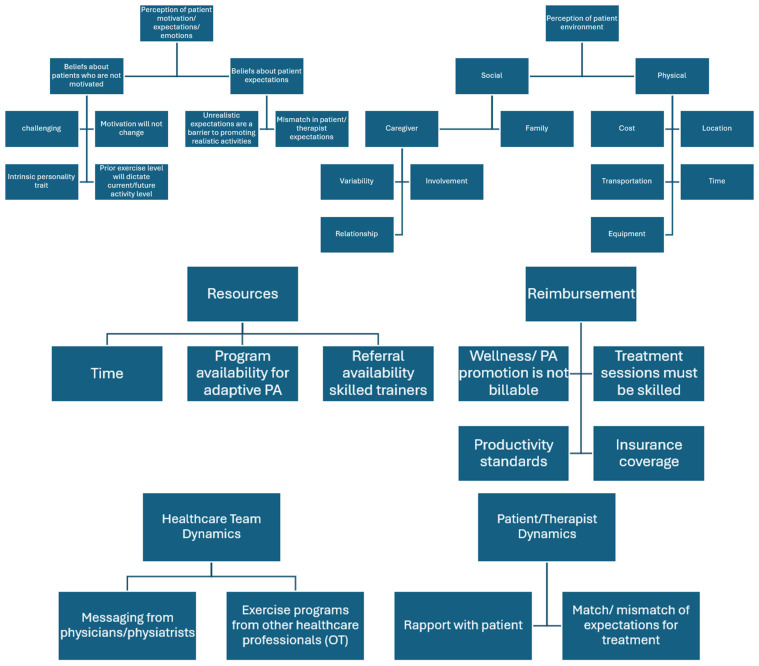
Coding trees for themes.

## Data Availability

The data presented in this study are available on request from the corresponding author. We chose not to make the data publicly available as the qualitative data could lead to possible identification of our study subjects as the study site location was included in the article.

## Abbreviations

The following abbreviations are used in this manuscript:

PA	Physical Activity
PT	Physical Therapist
BCT	Behavior Change Technique
SRQR	Standards for Reporting Qualitative Research
TDF	Theoretical Domains Framework
COM-B	Capability, Opportunity, and Motivation for Behavior Model
NCS	Board-Certified in Neurologic Physical Therapy
MS	Multiple Sclerosis
PD	Parkinson’s Disease
SCI	Spinal Cord Injury
AD	Assistive Device
PCA	Personal Care Assistant

## Appendix B. Interview Guide and Script

Introduction:

“Hello, and thank you for taking the time to participate in this interview and for letting me observe your sessions. I really appreciate your time. Today, I would like to talk more about the sessions I observed and get your insight into the discharge planning process and how you came to the decisions you made regarding activity planning. While I have a set of guiding questions, this interview is semi-structured, which means we can adapt the conversation based on your responses. Feel free to share any thoughts or examples that come to mind.

This interview will take approximately 45 min, and your responses will be anonymized. With your permission, I’d like to record our conversation to ensure I capture your responses accurately.

Do you have any questions before we begin? And is it okay to start the recording now?”

BCT use

▪ Have you ever heard of behavior change strategies?
▪ Have you ever tried any strategies to promote carryover and behavior change (if they have heard of it, can also frame as motivation) in your practice? (For example, action planning, goal setting, etc.)
  - What do you do? Why or why not? Follow up based on responses here
▪ What is your sense of carryover based on the conversations you have? Do you track it?
▪ What do you think are biggest limitations to your patients doing PA after discharge?
▪ Do you feel that PT has a role in addressing any of these limitations?
▪ Do you think that using these techniques would have any impact on PA?
▪ I saw you used XX strategy/approach, what made you choose this approach? What made you choose XX approach with this patient specifically?
▪ How do you choose with patients if you will do this?
▪ Have you done any other approaches? If so, what?
▪ What other factors do you weigh?
▪ How often do you incorporate behavior change techniques into your practice? When do you typically start doing them within your plan of care?
▪ Where did you learn about behavior change techniques? (additional training, cont ed, etc.?)
▪ How do you feel about using these techniques?
▪ How do you feel about others using these techniques?
▪ Is there anything that would make doing this more often easier? Harder?
  - Do you document these techniques? Do you bill for them?
▪ What are your thoughts on the impact of using behavior change techniques in practice?
  promotion of physical activity
  - What are your priorities for this patient to take away at discharge after your plan of care?
  - I saw that you talked about (certain aspects of physical activity) with patient XX, what made you decide to bring this up?
    ○ Did you plan on having this conversation going into the session?
  - How do you think your patient responded to the conversation?
    ○ Have you talked to this patient about PA before?
    ○ Do you think XX will continue a PA plan after discharge? Why or why not?
    ○ What do you think your role is in XX activity level post d/c?
    ○ How do you typically approach physical activity conversations throughout the plan of care?
    ○ Do you plan on doing any follow up on this conversation?
    ○ Do you assess barriers/facilitators to physical activity in the home and community during your plan of care?
    ○ What is the typical response you get from your patients when you bring PA up?
  - I saw that you chose to talk to XX about PA but not YY, can you talk to me about why?
    ○ What do you think makes some patients better candidates for PA outside of the clinic?
    ○ What factors do you consider when you promote PA?
  - What do you think the PT role is in promoting physical activity after discharge in xx population?
  - What resources do you rely on to help promote PA?
    ○ What resources do you wish you had?
    ○ Do you think insurance provides sufficient resources and reimbursement to support this part of your treatment?
  - Do you ever use any guidelines for physical activity conversations and exercise? How did you learn about any guidelines?
  - How do you track/measure PA that happens outside of the clinic?
  - Did you talk about any activity plans in this patient’s discharge? If so, what?
▪ Are there any diagnoses that you are more or less likely to emphasize PA with? How about specific subpopulations (ie chronic or subacute?)
▪ Are there any health conditions that make you less likely to promote PA?
▪ Are there any environmental factors you consider?
▪ Any personal factors?
  - What specific barriers/facilitators to PA does this patient have in your opinion?
    ○ If no, what other types of exercises and activities do you typically put into a home program at discharge?
    ○ Is physical activity included? Why or why not?
  - What are the barriers you see to your patients staying active after d/c?
    ○ Is there anything that you think PTs can address?
    ○ Is there anything that you think would help PTs be able to better address this barrier (resources, environmental changes, etc.)?
  - What do you think PT role in health promotion is?
